# Notes and Notices

**Published:** 1895-03

**Authors:** 


### NOTES AND NOTICES.

DR. E. L. COLBURN has removed from Fremont, Nebraska, to Salt Lake City, Utah.

DR. M. D. SATTERLEE has removed from Chattanooga, Tenn., to Andover, Ohio.

DR. THEODORE ENGELBACH, long and favorably known as a reliable homœopathic pharmacist, in New Orleans, has sold out his interest to Mr. Aug. C. Freitag. Dr. Engelbach will hereafter confine himself to the practice of medicine, at 828 Canal Street, New Orleans, La.

SOUTHERN HOMŒOPATHIC MEDICAL ASSOCIATION.—The next regular meeting of the Southern Association will be held in the city of St. Louis, in November, 1895. The meetings of this association have been growing in interest, value, and attendance, until now they rank next to the meetings of the American Institute. Officers: Wm. C. Richardson, M. D., President, St. Louis, Mo.; W. W. French, M. D., First Vice-President, Chattanooga, Tenn.; Sarah J. Millsop, M. D., Second Vice-President, Bowling Green, Ky.; C. R. Mayer, M. D., Recording Secretary, New Orleans, La.; Lizzie Gray Gutherz, M. D., Corresponding Secretary, St. Louis, Mo.; A. M. Duffield, M. D., Treasurer, Huntsville, Ala.

COOK COUNTY HOSPITAL, Chicago, has the following names on its homœopathic staff for the year 1895: Charles Adams, E. H. Pratt, H. R. Chislett,

M. B. Blouke, and L. D. Rogers, surgeons; Charles Gatchell, T. E. Roberts, E. E. Reininger, W. S. White, and O. F. Pierce, physicians; and W. C. Willard, C. M. Beebe, John W. Streeter, gynecologists.

"THE HOMŒOPATHIC MEDICAL SOCIETY OF CHICAGO is still flourishing," writes Dr. L. D. Rogers, editor of *The People's Health Journal.* "Notwithstanding the blizzard last Wednesday night, fifty physicians attended the meeting. Dr. E. S. Bailey read a paper on 'Non-Hemorrhagic Uterine Discharges.' He stated that during the past three years he had made cultures from the discharges of several hundred patients. He exhibited a number of interesting bacteriological specimens in test tubes. The paper was particularly valuable on account of the original work which it reported.

"The subject for the next meeting, which will be held the first Wednesday evening in March, will be the 'Treatment of Malignant Growths.' A paper will be read on the 'Treatment by Antitonine.'"

THE MARKHAM SANATORIUM, located at the far-famed city of Marquette, Michigan, on the shores of Lake Superior, is commended to the attention of the profession. The Sanatorium is conducted on the cottage plan, patients having rooms away from the institution proper, and coming to it for meals and treatment, except when essential or desirable to have meals and rooms at the same place. This plan will readily commend itself to all sensitive patients, as large numbers of sick people are not congregated together. A special feature is made of home cooking, home service, and a home feeling. The medical staff is composed of Russel Case Markham, M. D., Mary Louise Markham, M. D., Superintendents; L. Paul Anderson, M. D., Mechano-Hydro and Electro-therapeutist; Martha Stark, Head Nurse, and a distinguished corps of consulting physicians in Chicago and Detroit.

THE HOMŒOPATHIC MEDICAL SOCIETY OF OHIO will hold its thirty-fourth annual session in Cleveland, Ohio, about the 14th or 15th of May next. The Secretary, Dr. Thomas M. Stewart, 266 Elm Street, Cincinnati, Ohio, has published a circular urging the members to "make prominent the materia medica and therapeutics of our school."

THE NEW ENGLAND HAHNEMANN ASSOCIATION.—The first annual meeting was held in the hall of the Young Men's Christian Association, corner of Boylston and Berkeley Streets, Boston, Mass., on Monday, January 14th, at 12 M., for the election of officers for the ensuing year, and the transaction of such other business as came before the meeting. Reports and addresses upon the objects of the Association were given.

The object of this Association is to aid in the support of Boston University School of Medicine, and the institutions intimately connected therewith and essential to its work.

All physicians are invited to become members, with the avowed object as stated, the annual dues being two dollars. For further particulars, apply to Dr. I. T. Talbot, Dean of the Faculty of Boston University School, 685 Boylston Street, Boston, Mass.

